# (Invited) Influence of Nd^3^⁺ Doping and Thermal Annealing on Luminescent Properties and Thermal Sensing of Na₂Ti₆O₁₃ Nanocrystals

**DOI:** 10.1002/asia.202401699

**Published:** 2025-05-06

**Authors:** Wesley S. Silva, Wagner F. Silva, Uéslen Rocha, Daiane M. Medeiros, Rayssa J. B. Motta, Nelson G. C. Astrath, Noelio. O. Dantas, Anielle C. A. Silva, Carlos Jacinto

**Affiliations:** ^1^ Nano‐Photonics and Imaging Group Institute of Physics Universidade Federal de Alagoas Maceió‐AL 57072–900 Brazil; ^2^ Laboratório de Microscopia Eletrônica de Transmissão (LabMET) Institute of Physics Universidade Federal de Alagoas Maceió‐AL 57072–900 Brazil; ^3^ Department of Physics Universidade Estadual de Maringá Maringá‐PR 87020–900 Brazil; ^4^ Laboratório de Novos Materiais Nanoestruturados e Funcionais Institute of Physics Universidade Federal de Alagoas Maceió‐AL 57072–900 Brazil

**Keywords:** Biological windows, Nanoparticles, Nanothermometry, Rare Earths, Relative thermal sensitivity, Thermal annealing

## Abstract

This study investigates the effects of Nd^3^⁺ doping and thermal annealing (at 250, 500, 650, and 800 °C) on the structural and luminescent properties of Nd^3^⁺‐doped Na₂Ti₆O₁₃ nanocrystals (NCs), with a focus on their potential for thermal sensing applications. The optimal doping concentration was found to be 0.5 wt% Nd^3^⁺, where luminescence intensity decreases with higher concentrations due to concentration quenching. Thermal annealing significantly enhances both the crystallinity and luminescence intensity of the NCs, with the most notable improvements observed up to 500 °C. However, heating beyond 650 °C induces a phase transition from Na₂Ti₆O₁₃ to TiO₂, which impacts the NCs' structural and luminescent properties. Thermal sensing performance was evaluated using the fluorescence intensity ratio (FIR) between emissions at 1060 nm and 1340 nm across a temperature range of 300–343K, revealing the highest relative thermal sensitivity (S_r_) of 3.28% K⁻¹ in the sample annealed at 250 °C. For applications requiring high emission intensity, the 0.5 wt% Nd^3^⁺‐doped Na₂Ti₆O₁₃ NCs annealed at 800 °C exhibited the highest figure of merit, combining high luminescence intensity at 1060 nm with excellent S_r_, making them ideal for nanothermometry. Notably, the excitation (808 nm) and emission wavelengths (900, 1060, and 1340 nm) fall within the biological tissue windows, suggesting significant potential for biological nanothermometry applications. This study underscores the critical role of optimizing both doping concentration and thermal annealing conditions to enhance the properties of NCs, offering new insights into their use for advanced thermal sensing applications in biological and medical fields.

## Graphical abstract

The effects of Nd^3^^+^ doping and thermal annealing on Na₂Ti₆O₁₃ nanocrystals are systematically studied with respect to their luminescent and thermal sensing properties. Optimal doping and annealing enhance crystallinity, emission intensity, and thermal sensitivity. Emission wavelengths within biological windows suggest promising applications in nanothermometry, particularly for temperature sensing in biological and medical environments.

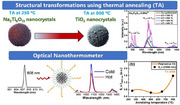

## 1 Introduction

Temperature measurement is crucial across various fields, including physics, biology, medicine, and materials research.^[^ ^1^, ^2^, ^3^, ^4^, ^5^, ^6^, ^7^, ^8^ ^]^ In nanomedicine, nanoparticles (NPs) have emerged as promising for both diagnostic and therapeutic applications in in vitro and in vivo.^[^ ^9^, ^10^, ^11^ ^]^ Real‐time temperature monitoring is particularly valuable in photothermal therapy, where it is essential to prevent overheating of healthy tissues.^[^ ^12^ ^]^ For effective anti‐cancer hyperthermia treatments, which selectively destroy cancer cells without damaging surrounding healthy tissue, maintaining local temperatures below 47 °C is critical.^[^ ^13^, ^14^ ^]^ However, achieving accurate temperature measurements within biological tissue remains a significant challenge.^[^ ^6^, ^15^, ^16^ ^]^ Non‐invasive techniques, such as magnetic resonance imaging (MRI), enable temperature mapping in biological tissues but require careful calibration specific to the tissue environment.^[^ ^17^, ^18^, ^19^ ^]^ Surface‐based methods like infrared thermography, however, only provide surface temperatures, which do not reflect internal tissue conditions.^[^ ^20^ ^]^

In recent years, luminescent nanothermometers (LNThs) have gained attention as an accurate and spatially resolved method for real‐time intra‐tissue temperature measurement.^[^ ^20^, ^21^, ^22^, ^23^ ^]^ These systems leverage temperature‐induced changes in luminescence properties, such as intensity, spectral position, band shape, and lifetime.^[^ ^24^ ^]^ Notably, near‐infrared (NIR) photoluminescence in the biological windows (BWs) has shown significant potential for biological studies at tissue depths from millimeters to centimeters, as scattering and absorption by biological tissues are minimized in these regions.^[^ ^25^, ^26^, ^27^, ^28^ ^]^ The three BWs are typically defined as 660–950 nm (I‐BW), 1000–1350 nm (II‐BW), and 1500–1800 nm (III‐BW).

The ratiometric method, which compares intensity changes between two temperature‐dependent emission bands, is particularly useful for thermometric applications. This approach mitigates for variations in pump intensity or fluctuations in emission due to changes in the nanothermometer concentration during measurements. For such applications, trivalent lanthanide (Ln^3^⁺)‐doped nanomaterials have attracted considerable research interest as LNThs due to their temperature‐sensitive luminescence properties. Among rare‐earth (RE)‐doped NPs, Nd^3+^‐doped materials are particularly popular. Nd^3+^ ions are advantageous because they can be excited at 800 nm (within the I‐BW), emitting at 900 nm (^4^F_3/2_ → ^4^I_9/2_), 1060 nm (^4^F_3/2_ → ^4^I_11/2_), and 1340 nm (^4^F_3/2_ → ^4^I_13/2_), which correspond to the I‐BW and II‐BW, respectively.^[^ ^29^ ^]^ Additionally, the 800 nm excitation wavelength is considered “heating‐free” as it is minimally absorbed by water. Despite all this positive information about this ion, previous studies on Nd^3^⁺‐doped nanomaterials have shown relatively low relative thermal sensitivity (S_r_), typically below 0.5% K⁻¹.^[^ ^20^, ^30^, ^31^ ^]^ This ion has very recently received attention as a sensitizer for many other ions. An interesting review paper on the Nd^3+^ potential in LNThs was published by Matulionyte et al.^[^ ^30^ ^]^

In relation to host matrices, sodium titanate (Na_2_Ti_6_O_13_) NPs have received significant attention in biomedical and technological fields due to their unique and versatile properties.^[^ ^32^, ^33^, ^34^, ^35^, ^36^ ^]^ Applications include bioactive ceramics,^[^ ^37^ ^]^ bone regeneration,^[^ ^38^ ^]^ and their potential as anode intercalation materials,^[^ ^39^ ^]^ with performance reviewed by Wu et al. in 2015.^[^ ^40^ ^]^ This material has also intrigued researchers for over two decades due to its pronounced metal‐to‐metal structural transition.^[^ ^41^, ^42^, ^43^, ^44^ ^]^ However, to date, there are no reports in the literature on Na_2_Ti_6_O_13_ nanocrystals (NCs) doped with rare‐earth (RE) ions. To the best of our knowledge, this is the first study reporting Na_2_Ti_6_O_13_ NCs doped with Nd^3^⁺ ions.

In this work, we explore the potential of Nd^3+^‐doped Na_2_Ti_6_O_13_ NCs for single‐band nanothermometry, with a particular focus on the impact of annealing temperature (AT) on their structural and optical properties, and also on S_r_. We examine four Nd^3+^ concentrations (0.5, 1.0, 5.0, and 10.0 wt%) and subject the samples to thermal annealing at 250, 500, 650, and 800 °C for 1 h. The results demonstrate that these NCs can serve as highly effective LNThs, achieving S_r_ values that surpass those of previously reported single‐core Nd^3^⁺‐doped systems.

## 2 Experimental Section

### 2.1 Synthesis of the Nanoparticles

The Na_2_Ti_6_O_13_ NCs were synthesized following a modified version of the method by Silva et al.^[^ ^45^ ^]^ A solution of 300 mL ultrapure water, 90 mL nitric acid (HNO_3_, 70%), urand 60 mL titanium isopropoxide (Ti(OCH(CH_3_)_2_)_4_, 97%) was prepared, with the pH adjusted to 12 using 4 m sodium hydroxide (NaOH, 98%). All reagents were obtained from Sigma‐Aldrich. This solution was left undisturbed to precipitate Na_2_Ti_6_O_13_ NCs. For Nd^3+^‐doped Na_2_Ti_6_O_13_ NCs (Na_2_Ti_6_O_13_:Nd^3+^), four concentrations of Nd^3^⁺ ions (0.5, 1.0, 5.0, and 10.0 wt%) were incorporated during the synthesis. The resulting precipitate was dispersed in ultrapure water and centrifuged at 6000 rpm for 10 min. Finally, the purified precipitate was subjected to successive thermal annealing in ambient atmosphere at 250, 500, 650, and 800 °C, with each temperature held for 1 h. All experiments performed in the present work were with samples in powder form.

### 2.2 Experimental Apparatus

The samples were excited by a continuous‐wave (CW) diode laser (Lumics) operating at 808 nm, focused directly onto a quartz cuvette containing the samples for fluorescence experiments. A typical excitation power (density) used in experiments was 1.5 W (∼80 kW/cm^2^). Heater plates attached to the sides of the cuvette, electrically controlled by an external voltage source, maintained the desired temperature for determining emissions of the Na_2_Ti_6_O_13_:Nd^3+^ NCs. This custom‐built temperature controller operated from room temperature up to 80 °C, with an estimated stability better than 1 °C. The fluorescence signal was collected using a NanoLog fluorimeter (HORIBA), equipped with an R5509‐73 photomultiplier tube cooled by liquid nitrogen, providing a detection range of 300–1700 nm. Transmission Electron Microscopy (TEM) images were obtained using a Tecnai G2 Spirit TWIN microscope at 120 kV.

## 3 Results and Discussions

**Figure 1:**
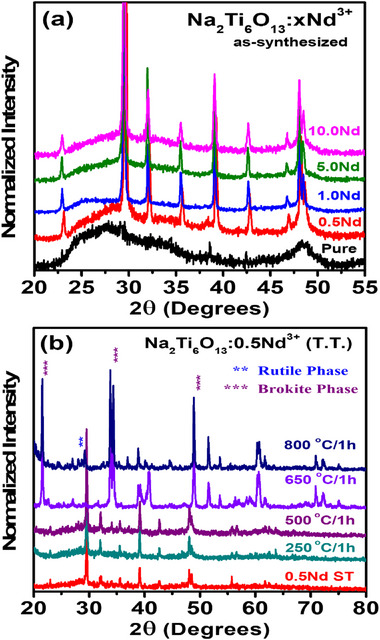
XRD patterns of (a) pure Na_2_Ti_6_O_13_ and Nd^3+^ doped samples (Na_2_Ti_6_O_13_:xNd^3+^) at concentrations of 0.5, 1.0, 5.0, and 10.0 wt% in the as‐synthesized form; (b) Na_2_Ti_6_O_13_:0.5Nd^3^⁺ as‐synthesized and after thermal annealing at 250, 500, 650, and 800 °C for 1 h. Crystalline phases are indicated by asterisks in the XRD patterns according to the corresponding standard cards.

The formation of Na_2_Ti_6_O_13_:Nd^3+^ NCs, the impact of Nd^3+^ doping on structural properties, and the effect of AT were investigated using X‐ray diffraction (XRD) analysis (Figure 1). As shown in Figure 1a, the XRD pattern of the undoped sample corresponds to amorphous hydrated sodium titanate (JCPDS n° 47–0124). With increasing Nd^3+^ concentration, the formation of Na_2_Ti_6_O_13_:Nd^3+^ NCs (JCPDS no. 73–1398) was promoted.^[^ ^46^ ^]^ Moreover, the XRD patterns of the 0.5 and 1.0Nd samples displayed higher crystallinity, while increased dopant levels led to structural deformations, resulting in partial amorphization. No dopant‐related peaks or impurities, such as neodymium oxide, were detected.^[^ ^47^ ^]^

To examine the effect of AT on the structural properties of Na_2_Ti_6_O_13_:Nd^3+^ NCs, the XRD patterns of the sample doped with 0.5 wt% Nd^3+^ ions (Na_2_Ti_6_O_13_:0.5Nd^3+^) are presented in Figure 1b. In the XRD pattern of the as‐synthesized sample annealed up to 500 °C, a narrowing of the diffraction peaks was observed, indicating increased crystallinity and a pure phase of Na_2_Ti_6_O_13_. However, the annealing at 650 and 800 °C favored the transformation of Na_2_Ti_6_O_13_ to TiO_2_ NCs, consistent with previous reports.^[^ ^48^ ^]^ The TiO_2_ NCs displayed both brookite and rutile phases, with crystalline phases marked by asterisks in the XRD patterns. The influence of Nd^3+^ doping on the structural properties of TiO_2_ NCs has also been documented in the literature.^[^ ^49^, ^50^, ^51^, ^52^ ^]^

**Figure 2:**
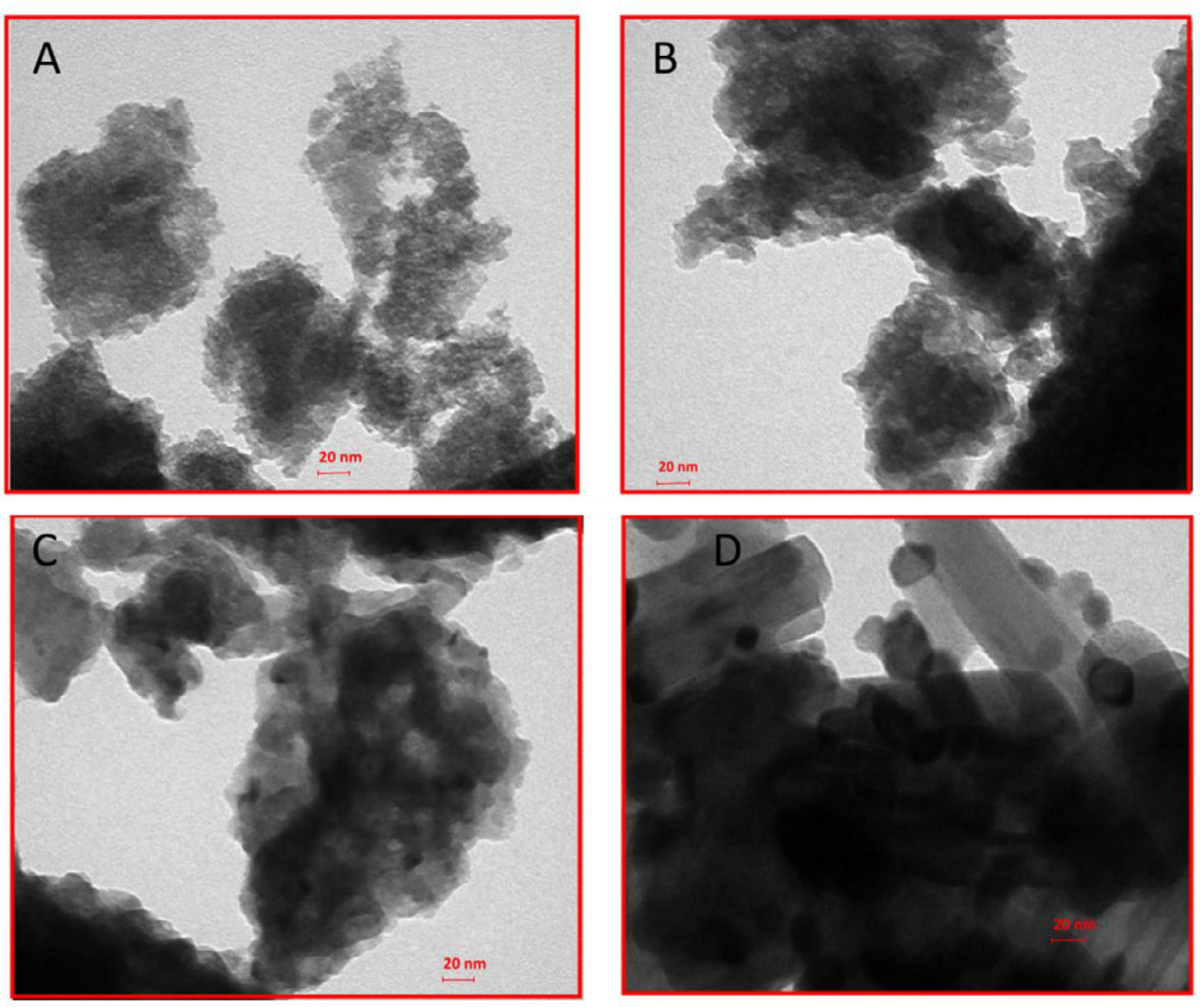
TEM micrographs of Na_2_Ti_6_O_13_ NPs subjected to thermal treatments at 250, 500, 650, and 800 °C. The images illustrate the morphological evolution of the NPs with increasing temperature, showing changes in aggregation, crystallinity, and particles´ size. Lower temperatures produce more amorphous and aggregated structures, while higher temperatures promote crystallization and grain growth.

The TEM micrographs in Figure 2 illustrate the morphological evolution of sodium titanate (Na_2_Ti_6_O_13_) NPs subjected to ATs at 250, 500, 650, and 800 °C. These images reveal subtle changes in particle morphology, crystallinity, and aggregation behavior as a function of temperature. The TEM images, shown in Panels a–d, demonstrate varying degrees of NPs aggregation and dispersion. Figure 2a,b depict TEM images for samples annealed at 250 and 500 °C, where the NPs appear as irregularly shaped clusters with significant agglomeration. This suggests strong interparticle interactions, which may be attributed to inadequate surface functionalization or high surface energy, promoting particle clustering. The absence of a surfactant during synthesis could further exacerbate these effects by preventing effective surface passivation.

In Figure 2c, a denser aggregation of NPs indicates increased particle‐particle interactions, which could negatively affect the particles’ dispersibility and stability in solution. The presence of larger clusters suggests that the NPs may have experienced Ostwald ripening or other growth mechanisms during synthesis. In contrast, Figure 2d shows a mixture of spherical and rod‐like NPs, implying anisotropic growth behavior. The rod‐shaped particles suggest directional crystallization, possibly influenced by synthesis factors such as reaction time, temperature, or precursor concentration.

These observations underscore the influence of thermal treatment on the structural evolution of Na_2_Ti_6_O_13_ NPs. At lower temperatures, the particles exhibit an amorphous and aggregated structure, while higher temperatures promote crystallinity and particle growth, leading to improved stability but potentially reducing surface area. These findings highlight the need for optimizing thermal treatment conditions to tailor material properties for specific applications. Further studies will focus on fine‐tuning these parameters to achieve consistent and reproducible NP characteristics.

**Figure 3:**
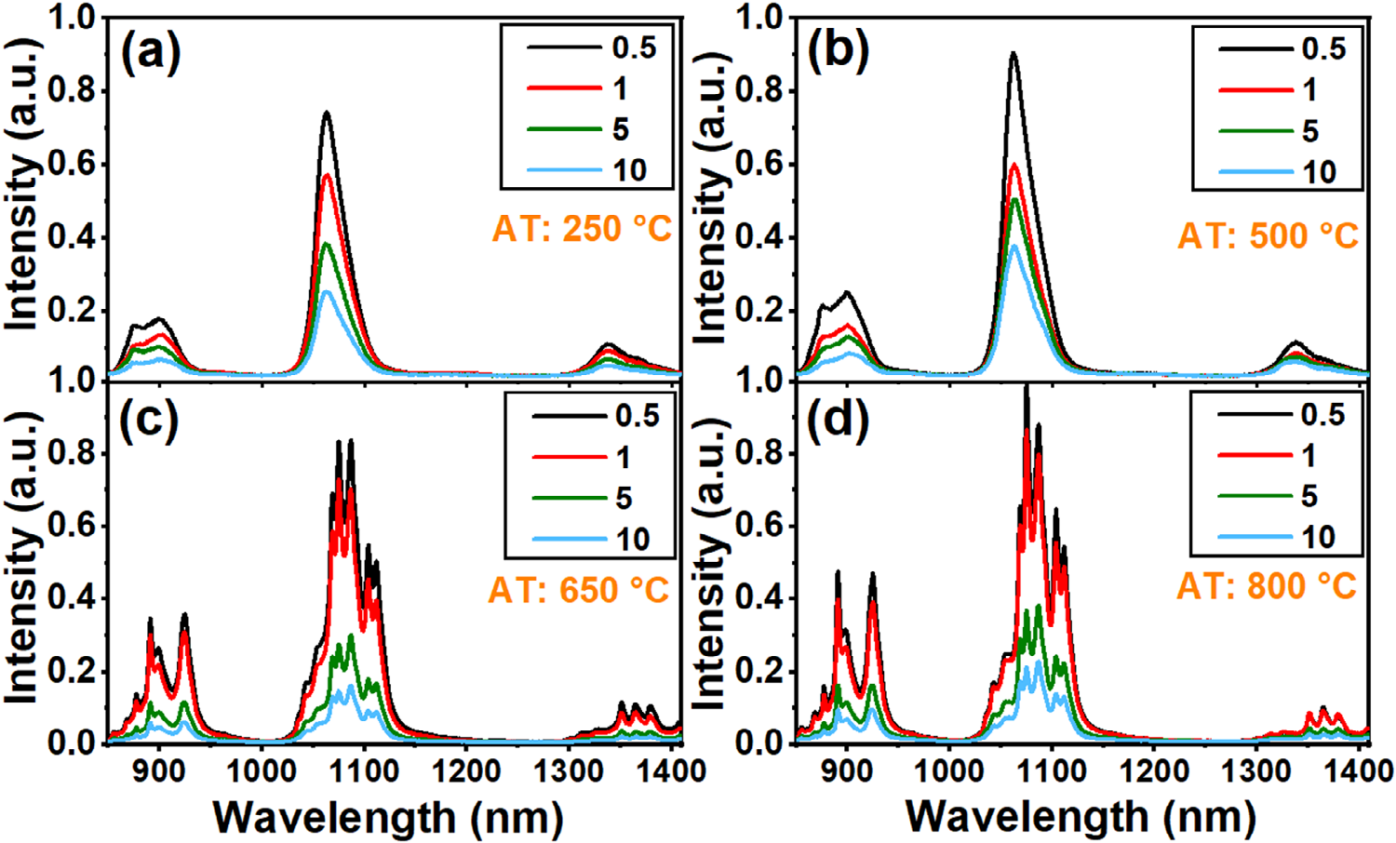
Emission spectra of Na_2_Ti_6_O_13_:xNd^3+^ NCs (x = 0.5, 1.0, 5.0, and 10.0 wt%) at four different annealing temperature (AT): (a) 250, (b) 500, (c) 650, and (d) 800 °C, under 808 nm laser excitation.

**Figure 4:**
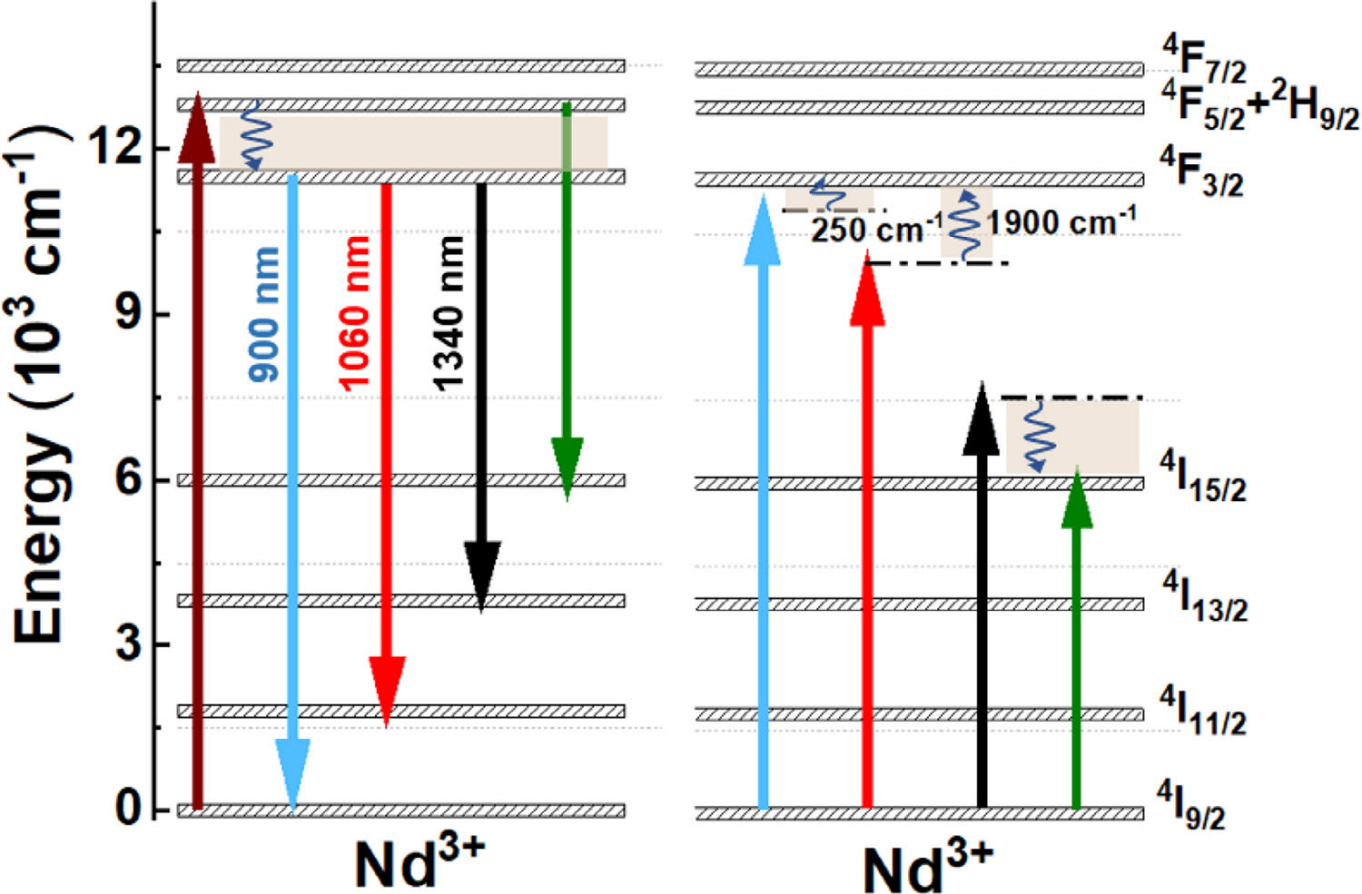
Simplified energy level diagram of the Na_2_Ti_6_O_13_:Nd^3+^ system, illustrating possible cross‐relaxation processes. The diagram includes absorption at 808 nm (upward arrow), emission pathways (downward arrows), and multiphonon relaxation processes (wavy arrows).

Figure 3 shows emission spectra of Na_2_Ti_6_O_13_:xNd^3+^ NCs (x = 0.5, 1.0, 5.0, and 10.0 wt%) annealed at the four temperatures (250, 500, 650, and 800 °C) under 808 nm laser excitation. The spectra reveal three distinct Nd^3+^ emissions around 900 nm (^4^F_3/2_ → ^4^I_9/2_), 1060 nm (^4^F_3/2_ → ^4^I_11/2_), and 1340 nm (^4^F_3/2_ → ^4^I_13/2_). Upon 808 nm excitation, Nd^3+^ ions are initially excited to the ^4^F_5/2 _+ ^2^H_9/2_ levels, followed by rapid multiphonon relaxation to the ^4^F_3/2_ state, from which these emissions originate (Figure 4). Notably, both the excitation and the observed emissions (900, 1060, and 1340 nm) fall within the BWs of human tissues, where light absorption and scattering are minimized, allowing partial transparency at these wavelengths.^[^ ^8^, ^27^, ^28^ ^]^ Therefore, these NCs hold promise for biological applications. As shown in Figure 3a, the luminescence intensity of Na_2_Ti_6_O_13_:xNd^3+^ NCs annealed at 250 °C decreases as the Nd^3^⁺ ion concentration increases. This trend is consistent across NCs subjected to thermal annealing at 500, 650, and 800 °C, as illustrated in Figure 3b–d.

Figure 3 also shows that the crystallinity of the NCs improves with higher ATs, which in turn enhances the luminescence emissions. Notably, at elevated ATs (650 and 800 °C), peak splitting of the luminescence emissions is observed. This phenomenon is attributed to the Nd^3+^ ions being situated within a more organized crystalline lattice, specifically TiO_2_, as opposed to Na_2_Ti_6_O_13_. For instance, at 250 °C, a single emission peak is observed around 1060 nm, while at 650 and 800 °C, multiple peaks emerge around this wavelength.

Numerous studies in the literature have reported high fluorescence quantum efficiencies at low Nd^3^⁺ ion concentrations (1 to 5 mol%),^[^ ^53^, ^54^ ^]^ although other research also demonstrates efficient NIR emissions at higher neodymium concentrations (up to 10%).^[^ ^55^ ^]^ It is well established that high Nd^3^⁺ ion concentrations can lead to reduced luminescence due to concentration quenching and can cause non‐negligible heating effects.^[^ ^22^, ^56^ ^]^ This quenching phenomenon is common in lanthanide‐doped systems, particularly with Nd^3^⁺ ions, and occurs because increasing ion concentration reduces the average distance between Nd^3^⁺ ions. When the distance falls below a critical threshold‐dependent on factors such as the host material, particle size, and ion distribution—non‐radiative processes become more probable. Specifically, cross‐relaxation between closely spaced ions diminishes the likelihood of radiative transitions, thereby lowering emission intensity.^[^ ^57^, ^58^ ^]^ Consequently, there is no universally agreed‐upon optimal concentration of Nd^3^⁺ ions, as this depends largely on the intended application. For example, an optimal ion concentration is necessary for fluorescence imaging, where brightness is defined as the product of fluorescence quantum efficiency and optimal ion concentration.^[^ ^54^ ^]^ In our study, since the goal is to use the NCs as nanothermometers, high luminescent emission is preferred. Therefore, the optimal concentration of Nd^3^⁺ ions for the samples examined here is 0.5 wt%. This concentration was chosen to investigate the influence of thermal annealing on relative thermal sensitivity, which will be discussed in the following section.

**Figure 5:**
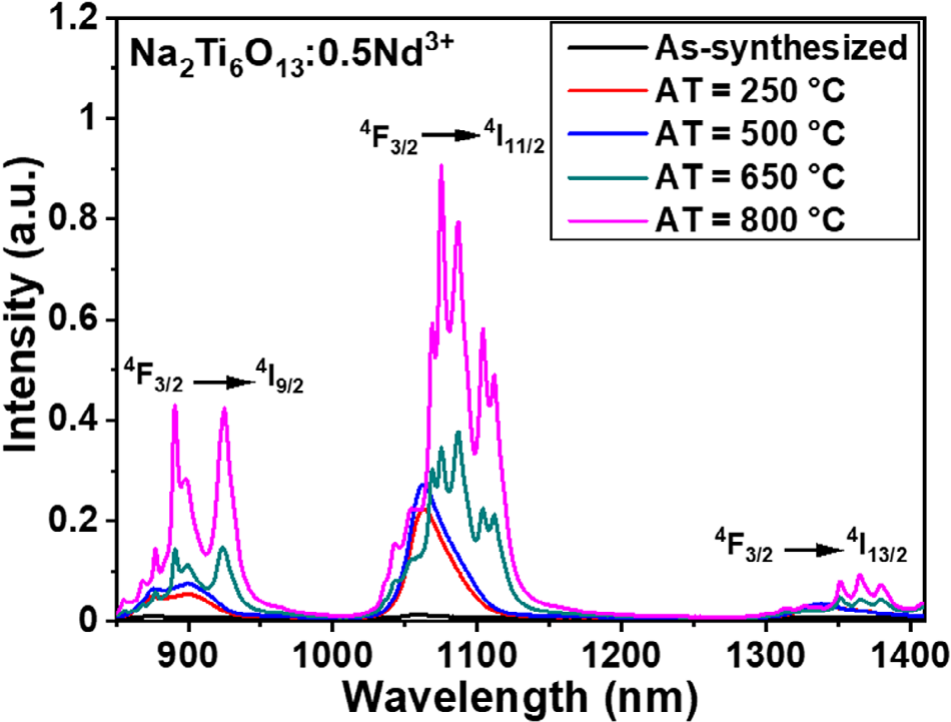
Emission spectra of as‐synthesized Na_2_Ti_6_O_13_:0.5Nd^3+^ NCs, along with samples annealed at four different temperatures (250, 500, 650, and 800 °C), under 808 nm excitation.

Figure 5 depicts the emission spectra of Na_2_Ti_6_O_13_:0.5Nd^3+^ NCs, excited at 808 nm, both as‐synthesized and after ATs of 250, 500, 650, and 800 °C. The data reveal that luminescence emission increases with higher ATs. In particular, the Na_2_Ti_6_O_13_:0.5Nd^3+^ NCs annealed at 800 °C exhibit the highest luminescence intensity compared to the other samples. This enhancement is attributed to improved crystallinity and a reduction in surface defects, which minimizes non‐radiative decay, as previously reported for other lanthanide‐doped materials.^[^ ^57^ ^]^ Generally, thermal annealing facilitates particle growth by promoting atomic diffusion between smaller particles.

A system comprising smaller particles has a greater number of grain boundaries compared to larger particles. This results in more dangling bonds and disorder in the atomic arrangement, which can lead to quenching of luminescence due to non‐radiative losses, such as multiphonon relaxation. As the AT increases, the atomic arrangement becomes more organized, and dangling bonds are likely diminished. While larger NPs may provide more Nd^3+^ ions available on their surfaces, thermal annealing typically promotes the diffusion of lanthanide ions from the surface to the core of the NPs.^[^ ^57^, ^59^ ^]^ In the case of core‐shell structures, ions may diffuse from the core to the shell. Consequently, the optimal optical properties of Nd^3+^ ions are observed when they are incorporated into a more organized crystal lattice, such as that found in TiO_2,_ in comparison with Na_2_Ti_6_O_13_.

**Figure 6:**
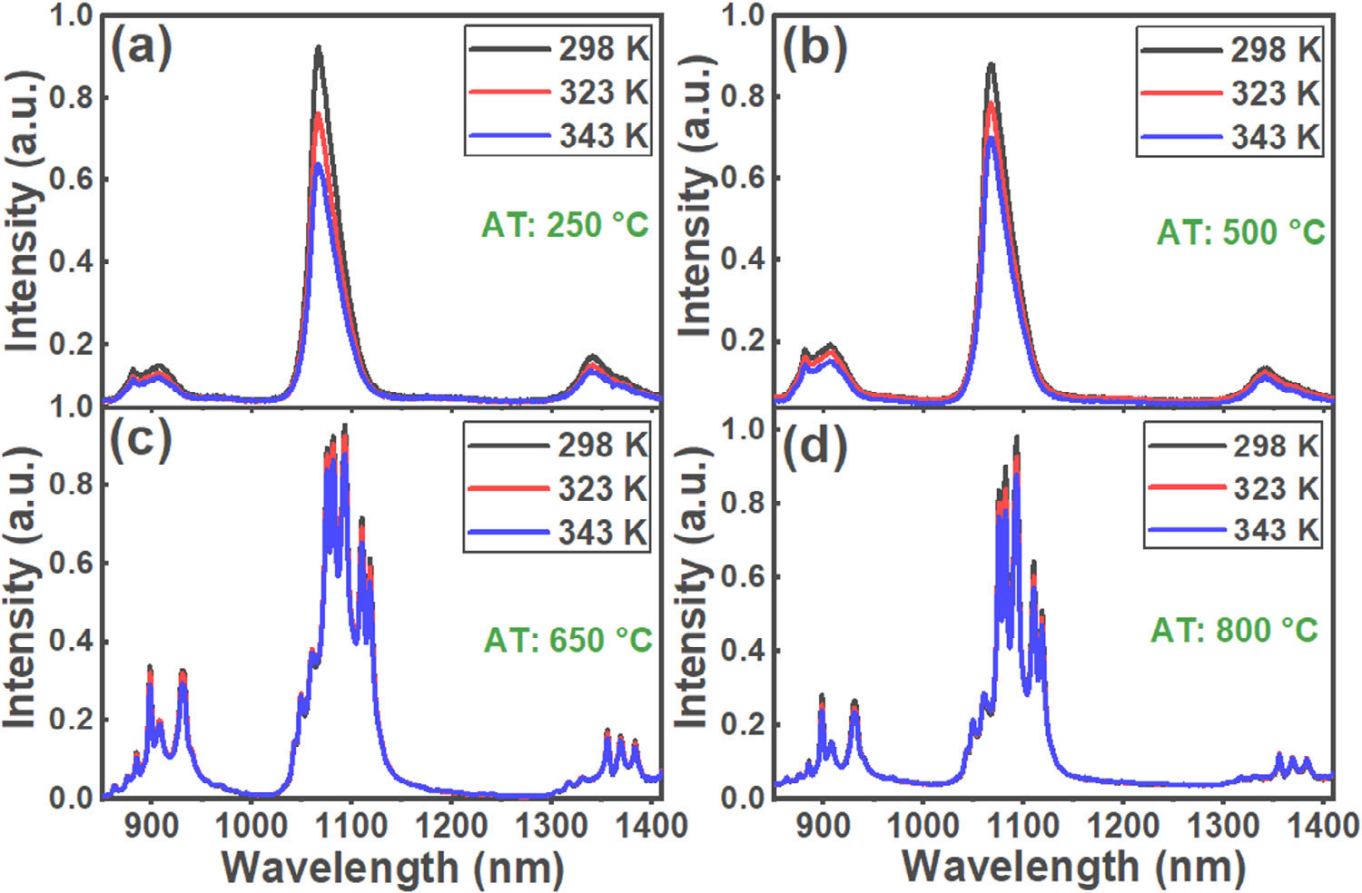
Emission spectra of Na_2_Ti_6_O_13_:0.5Nd^3+^ NCs recorded at three different temperatures (298 K, 323 K, and 343 K) for samples annealed at (a) 250, (b) 500, (c) 650, and (d) 800 °C, under 808 nm excitation. Emission intensity decreases with rising temperature, particularly at 900, 1060, and 1340 nm, corresponding to the transitions ^4^F_3/2_ → ^4^I_11/2_, ^4^F_3/2_ → ^4^I_11/2_, and ^4^F_3/2_ → ^4^I_9/2_, respectively, of Nd^3^⁺ ions.

The emission spectra of Na_2_Ti_6_O_13_:0.5Nd^3+^ NCs, excited at 808 nm, at three different temperatures (298 K, 323 K, and 343 K) following ATs at 250, 500, 650, and 800 °C are shown in Figure 6a–d. The emission intensity decreases with rising temperature across all three observed emission bands. This decline is attributed to the thermal coupling between the ^4^F_3/2_ and ^4^F_5/2 _+ ^2^H_9/2_ levels (as illustrated in Figure 4), alongside energy transfer (ET) processes (including cross‐relaxation and energy migration), which are likely to intensify with temperature. The energy gap (Δ*E*) between the ^4^F_3/2_ and ^4^F_5/2 _+ ^2^H_9/2_ levels is approximately 1100 cm⁻¹. This thermal coupling facilitates the transfer of population from the ^4^F_3/2_ level to the ^4^F_5/2 _+ ^2^H_9/2_ states as the temperature increases, thereby reducing the population of the ^4^F_3/2_ level and, consequently, its emission intensity.^[^ ^60^, ^61^, ^62^ ^]^

As shown in Figure 6, significant variations in emissions are associated with samples annealed at lower temperatures, corroborating the explanation put above on the reduced surface defects and the increase in the samples´ crystallinity. Additionally, emissions around 900 and 1060 nm are reduced more compared to that one at 1340 nm. This behavior probably results from the differing temperature dependencies of each cross‐relaxation (CR) process, as outlined in Figure 4.^[^ ^63^ ^]^ In this figure, the transitions near 900 nm [(^4^F_3/2_, ^4^I_9/2_) → (^4^I_9/2_, ^4^F_3/2_)+ΔE] and 1060 nm [(^4^F_3/2_, ^4^I_9/2_)→(^4^I_11/2_, ^4^F_3/2_) +ΔE] represent anti‐Stokes ET processes, while the 1340 nm transition [(^4^F_3/2_, ^4^I_9/2_)→(^4^I_11/2_, ^4^I_15/2_) ‐ ΔE] is a Stokes ET process. As is known, unlike Stokes processes, anti‐Stokes ET processes are highly temperature‐dependent and increase with it.^[^ ^64^, ^65^, ^66^, ^67^ ^]^

The suitability of a luminescent thermometer for temperature sensing is quantified by its relative thermal sensitivity (*S_r_*), which is defined as:

(1) $$S_{r}=\frac{1}{\Delta \left(T\right)}\left|\frac{\partial \Delta }{\partial T}\right|\times \;100\%$$

where Δ(T) is the thermometric parameter, e.g., the fluorescence intensity ratio (FIR) of selected emissions at a given temperature or as a function of the temperature (T). This sensitivity factor *S_r_* reflects the rate of change in Δ with respect to temperature, making it a crucial parameter in evaluating the thermometer accuracy and responsiveness for nanothermometry applications. As previously mentioned, the FIR method is independent of both sample quantity and excitation intensity, making it an exceptionally reliable tool for thermal sensing.

**Figure 7:**
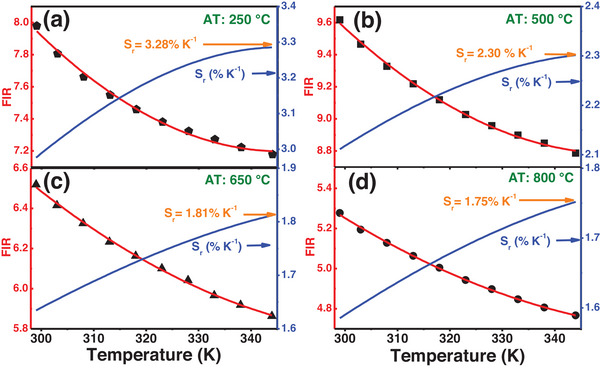
Fluorescence intensity ratio (FIR) of emissions at 1060 and 1340 nm (I_1060 nm_/I_1340 nm_) and curves of relative thermal sensitivity (*S_r_*) for Na_2_Ti_6_O_13_:0.5Nd^3+^ NCs corresponding to four different annealing temperatures (AT): (a) 250, (b) 500, (c) 650, and (d) 800 °C, under 808 nm excitation. Solid lines associated with the experimental FIR points represent polynomial fitting, capturing the trends in relative thermal sensitivity across varying annealing conditions.

In this study, the FIR for the emissions at 1060 and 1340 nm (FIR = I_1060 nm_/I_1340 nm_) under 808 nm excitation was calculated for Na_2_Ti_6_O_13_:0.5Nd^3+^ NCs subjected to ATs at 250, 500, 650, and 800 °C, as displayed in Figure 7. The corresponding *S_r_* curves are also presented as blue lines (left axis). To provide a smoother representation of the FIR data, solid red lines represent polynomial fits—commonly used for non‐thermally coupled energy levels—applied to the experimental FIR data. The polynomial fits are given for each AT condition as follows:

(2a) $${\mathrm{FIR}}_{250}=48.09-0.2365\;\times T+3.420\;\times 10^{-4}T^{2}$$

(2b) $${\mathrm{FIR}}_{500}=44.45-0.2026\;\times T+2.876\;\times 10^{-4}T^{2}$$

(2c) $${\mathrm{FIR}}_{650}=25.48-0.1063\;\times T+1.433\;\times 10^{-4}T^{2}$$

(2d) $${\mathrm{FIR}}_{800}=20.17-0.0835\;\times T+1.126\;\times 10^{-4}T^{2}$$

These polynomial fits capture the overall trend of the data, reinforcing the reliability of the FIR as a parameter for thermal sensing across different annealing conditions. The FIR fits were also used to determine the corresponding *S_r_* curves. The maximum *S_r_* values were found to be 3.28%, 2.30%, 1.81%, and 1.75 % K^−1^ for ATs of 250, 500, 650, and 800 °C, respectively. Notably, *S_r_* decreases as the AT increases, which can be attributed to the enhanced crystallinity and the phase transformation from Na_2_Ti_6_O_13_ to TiO_2_ NCs at higher ATs, as discussed earlier.

Table 1 shows a comparison of S_r_ values for different luminescent thermometers (LThs) that utilize the FIR method. The maximum S_r_ value obtained in the current study is among the highest reported in the literature for Nd^3+^ doped NPs. Typically, these values fall below 3.0% K^−1^. For instance, previous studies reported the following S_r_: 0.25%K^−1^ (LaF_3_:Nd^3+^),^[^ ^20^ ^]^ 0.3%K^−1^ (LiLaP_4_O_12_:Nd^3+^,Yb^3+^),^[^ ^68^ ^]^ 1–2%K^−1^ (NaYF_4_:Nd,Yb@NaYF_4_:Er,Yb),^[^ ^69^ ^]^ 2.5%K^−1^ (NaGdF_4_ + CdS/PbS/ZnS@PLGA:Nd^3+^),^[^ ^70^ ^]^ 1.49%K^−1^ (Lu_2_CaMg_2_Ge_3_O_12_:Yb,Nd),^[^ ^71^ ^]^ 1.25%K^−1^ (GdP_5_O_14_:Cr^3+^,Nd),^[^ ^72^ ^]^ 1.12%K^−1^ (NaYF_4_:Yb,Nd,Er@NaYbF_4_).^[^ ^73^ ^]^ Specifically, these last three mentioned studies focus on improving the S_r_ of LThs using various material strategies: Zhao et al.,^[^ ^71^ ^]^used a garnet oxide Lu_2_CaMg_2_Ge_3_O_12_ doped with Yb^3+^, Nd^3+^ and Er^3+^ ions, achieving S_r_ of up to 2.62% K^−1^ for Nd^3+^ and 1.15% K^−1^ for Er^3+^, enhanced by the strong crystal field effect; Maciejewska and Marciniak^[^ ^72^ ^]^ explored GdP_5_O_14_ NCs co‐doped with Cr^3+^ and Nd^3+^ ions, offering a multimodal temperature reading approach, with Mode II (590 nm/1060 nm) showing the best performance and S_r_; and Xu et al.,^[^ ^73^ ^]^ investigated NPs based on NaYF_4_ core‐shell structures, demonstrating that the combination of inert and active layers significantly increases upconversion luminescence and S_r_, with a higher S_r_ of 1.12% K^−1^. Together, these works highlight the potential of advanced materials designs to improve the performance of LThs. In relation to our study, one of the notable advantages of Na_2_Ti_6_O_13_:0.5Nd^3+^ NCs is their relatively simple structure, consisting of a single core doped solely with Nd^3+^ ions. This simplicity may contribute to their enhanced S_r_ and makes them appealing for potential applications in thermal sensing.

**Table 1:** Relative thermal sensitivity (S_r_) of various Nd^3^⁺‐doped NPs determined via the fluorescence intensity ratio (FIR) method.

System	Excitation Wavelength (nm)	Operating Wavelength (nm)	S_r_ [ × % K^−1^]	Ref.
Na_2_Ti_6_O_13_:0.5Nd^3+^ (AT: 250 °C)	808	1000–1400	3.28	This work
Na_2_Ti_6_O_13_:0.5Nd^3+^ (AT: 500 °C)	808	1000–1400	2.30	This work
Na_2_Ti_6_O_13_:0.5Nd^3+^ (AT: 650 °C)	808	1000–1400	1.81	This work
Na_2_Ti_6_O_13_:0.5Nd^3+^ (AT: 800 °C)	808	1000–1400	1.75	This work
LaF_3_:Nd	808	850–930	0.26	^[^ ^20^ ^]^
LiLaP_4_O_12_:Nd^3+^,Yb^3+^	808	850–1100	0.30	^[^ ^68^ ^]^
NaYF_4_:Nd,Yb@NaYF_4_:Er,Yb	808	500–550 and 850–1100	2.00	^[^ ^69^ ^]^
NaGdF_4_ + CdS/PbS/ZnS@PLGA:Nd^3+^	808	1000–1300	2.50	^[^ ^70^ ^]^
YVO_4_:Nd	808	1063	0.25	^[^ ^74^ ^]^
YAG:Nd	808	940	0.15	^[^ ^75^ ^]^
NaYF_4_:Nd	830	870	0.12	^[^ ^76^ ^]^
LaF_3_:Nd@LaF_3_:Yb	790	1000–1330	0.41	^[^ ^29^ ^]^
Lu_2_CaMg_2_Ge_3_O_12_:Yb,Nd	980	756/805	1.49	^[^ ^71^ ^]^
GdP_5_O_14_:Cr^3+^,Nd	808	590/1060	1.25	^[^ ^72^ ^]^
NaYF_4_:Yb,Nd,Er@NaYbF_4_	980	526/540	1.12	^[^ ^73^ ^]^

The respective spectral operational ranges are also indicated.

**Figure 8:**
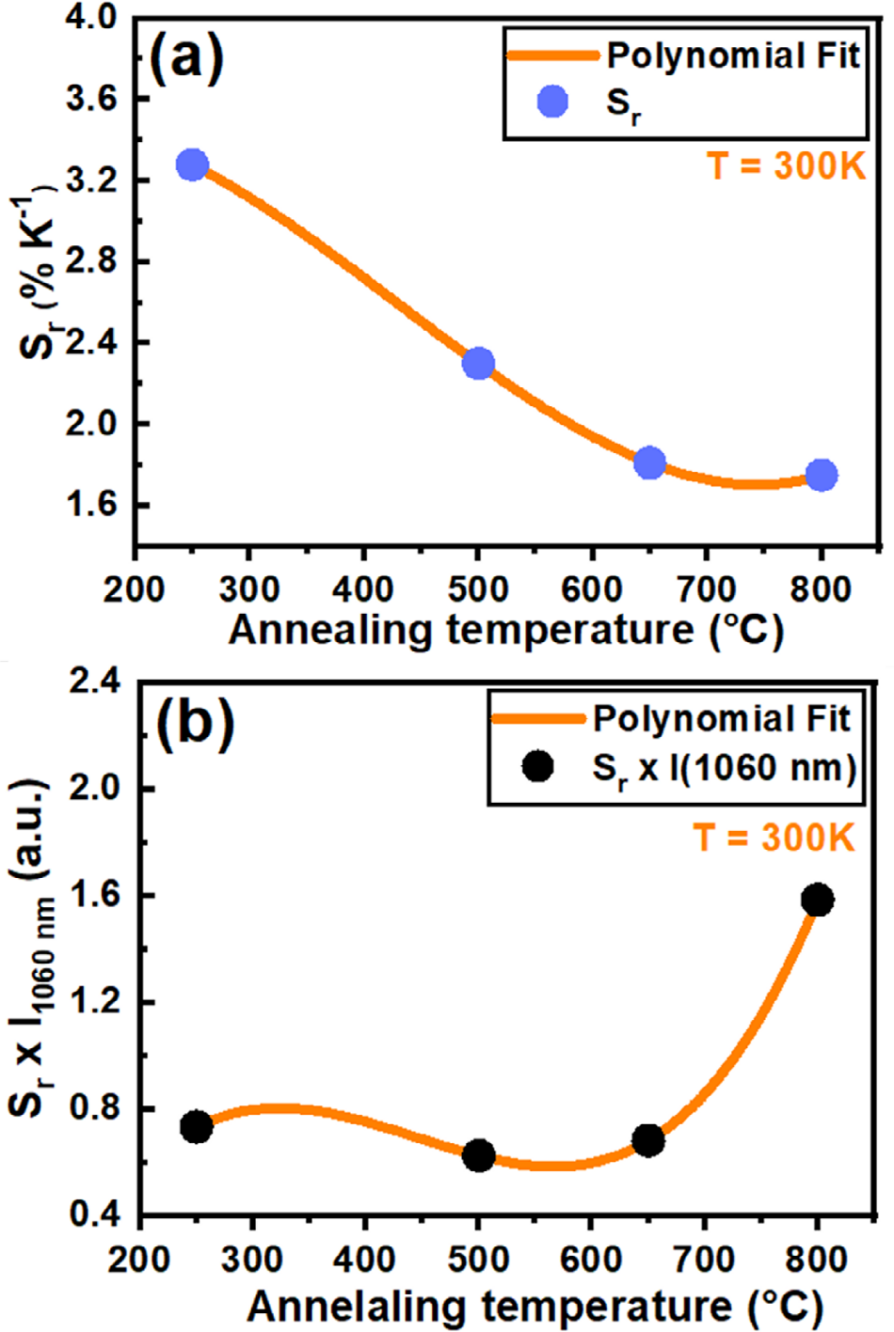
(a) Relative thermal sensitivity (*S_r_*) at 300K and (b) product of relative thermal sensitivity and emission peak value at 1060 nm (*S_r_* × I_1060nm_) for Na_2_Ti_6_O_13_:0.5Nd^3+^ NCs as a function of annealing temperature. The curves connecting the data points serve as guides to the eye, illustrating the relationship between thermal sensitivity and annealing conditions.

As previously discussed, the Na_2_Ti_6_O_13_:0.5Nd^3+^ NCs annealed at 800 °C exhibit the highest luminescence. However, this sample shows the lowest S_r_ compared to the other samples studied, as shown in Figure 8a. Notably, this AT coincides with the transformation of Na_2_Ti_6_O_13_ to TiO_2_, which likely contributes to the observed decrease in S_r_. While Nd^3+^‐doped TiO_2_ NCs possess a more organized crystal lattice than those doped with Nd^3+^ in Na_2_Ti_6_O_13_, this structural change affects their performance as LThs. To more effectively assess the suitability of each sample as a LNTh for thermal imaging, we calculated the figure of merit, defined as the product of luminescence intensity and the maximum S_r_ value. This analysis is illustrated in Figure 7b, where the product of maximum Sr values (from Figure 8a) and the emission intensity at 1060 nm (*S_r_* x *I_1060 nm_*) is shown. This approach provides a more comprehensive evaluation of the sample performance, allowing for a clearer comparison of their potential for thermal sensing applications. For nanothermometry, it is crucial that the NPs not only exhibit high S_r_ values but also strong luminescent emissions.

In summary, as illustrated in Figure 8b, the Na_2_Ti_6_O_13_:0.5Nd^3+^ NCs annealed at 800 °C emerge as the most suitable option for use as a nanothermometer among the samples analyzed. However, if the primary focus is on achieving the highest maximum S_r_ (3.28% K^−1^), the Na_2_Ti_6_O_13_:0.5Nd^3+^ NCs annealed at 250 °C are the preferred choice, as indicated in Figure 8a. It is also worth noting that these NCs can be excited in the I‐BW (808 nm), with emissions used for temperature measurement occurring in the II‐BW (1060 and 1340 nm). Therefore, these NCs hold significant potential as nanothermometers for biological applications. This work also introduces a novel approach for synthesizing Nd^3+^‐doped TiO_2_ NCs via the transformation of Na_2_Ti_6_O_13_ to TiO_2_, with results that surpass those of previous studies.^[^ ^49^ ^]^ Another very important issue is the stability of the materials. In fact, stability is a critical factor that limits the practical application of nanomaterials, particularly in fields like nanothermometry, where consistent performance over time is essential, especially in biological environments. Although our study primarily focused on the luminescent and thermal sensing properties of Nd^3^⁺‐doped Na₂Ti₆O₁₃ NCs, we recognize the need for further investigation into their long‐term stability. Future work will include experiments to evaluate the chemical and thermal stability of the NCs under various environmental conditions, such as humidity, light exposure, and temperature cycles. Additionally, we plan to evaluate potential issues like ion leaching and phase degradation, particularly at elevated temperatures, which may impact their stability and performance. Such stability analyses will be crucial for understanding the material's suitability for real‐world applications, especially in biological nanothermometry.

## 4 Conclusions

This study investigates the impact of Nd^3^⁺ doping and thermal annealing on the structural and luminescent properties of Nd^3^⁺‐doped Na₂Ti₆O₁₃ nanocrystals (NCs), with a particular focus on their potential for thermal sensing applications. We found that the luminescence intensity of the NCs decreases with increasing Nd^3^⁺ concentration, and the optimal doping level for enhanced performance is 0.5 wt%. Thermal annealing improves the crystallinity and emission intensity of the NCs, with significant structural changes observed at higher annealing temperatures. Notably, the transition from Na₂Ti₆O₁₃ to TiO₂ above 650 °C correlates with changes in particle morphology and crystallinity. These transformations influence the luminescence behavior, with the 800 °C annealed NCs exhibiting the highest luminescence intensity, likely due to the more organized crystalline structure of TiO₂. Furthermore, the observed changes in particle size and shape, particularly at higher annealing temperatures, are crucial factors that contribute to the enhanced optical properties, as they reduce surface defects and increase the overall crystallinity. The highest relative thermal sensitivity (3.28% K⁻¹) was observed in the NCs annealed at 250 °C, while the combination of high emission intensity and thermal sensitivity (Sr × I₁₀₆₀ nm) was maximized in the 800 °C annealed samples, making them most suitable for nanothermometry applications. Additionally, the excitation and emission wavelengths fall within biological tissue windows, suggesting strong potential for biological nanothermometry. These findings highlight the importance of optimizing both thermal treatment conditions and particle morphology to tailor the properties of NCs for specific applications, especially in advanced thermal sensing and nanothermometry. Further investigations into the long‐term stability of these materials under various environmental conditions will be essential for evaluating their practical use, particularly in biological environments.

## Authors Contributions

W. Soares conducted the experimental work related to optical spectroscopy and nanothermometry. A. C. A. Silva and N. O. Dantas were responsible for synthesizing the nanocrystals and contributed to the discussion of the results. W. F. Silva participated in writing and analyzing the results. U. Rocha assisted with the experiments and contributed to the discussions. D. M. Medeiros, R. J. B. Motta were responsible for the experiments and structural analysis of the samples. N. G. C. Astrath participated in writing and analyzing the results. C. Jacinto led the research, overseeing its planning, discussing the results, and writing the manuscript.

## Conflict of Interests

The authors declare no conflict of interest

## Data Availability

The data that support the findings of this study are available from the corresponding author upon reasonable request.
